# Assessing the utility of artificial intelligence throughout the triage outpatients: a prospective randomized controlled clinical study

**DOI:** 10.3389/fpubh.2024.1391906

**Published:** 2024-05-30

**Authors:** Xiaoni Liu, Rui Lai, Chaoling Wu, Changjian Yan, Zhe Gan, Yaru Yang, Xiangtai Zeng, Jin Liu, Liangliang Liao, Yuansheng Lin, Hongmei Jing, Weilong Zhang

**Affiliations:** ^1^Department of Hematology, Lymphoma Research Center, Peking University Third Hospital, Beijing, China; ^2^Department of Respiratory Medicine, First Affiliated Hospital Gannan Medical University, Ganzhou, China; ^3^Department of Respiratory Medicine, The People's Hospital of Ruijin City, Ruijin, China; ^4^Department of Respiratory Medicine, Affiliated Hospital of Jiujiang University, Jiujiang, China; ^5^The Second Affiliated Hospital of Fujian Medical University, Quanzhou, China; ^6^Gannan Medical University, Ganzhou, China; ^7^Department of Thyroid and Hernia Surgery, First Affiliated Hospital of Gannan Medical University, Ganzhou, Jiangxi, China; ^8^Department of Respiratory Medicine, Longnan First People's Hospital, Longnan, China; ^9^Department of Emergency and Critical Care Medicine, Suzhou Hospital, Affiliated Hospital of Medical School, Nanjing University, Suzhou, China

**Keywords:** artificial intelligence, ChatGPT, triage outpatients, AI, triage

## Abstract

Currently, there are still many patients who require outpatient triage assistance. ChatGPT, a natural language processing tool powered by artificial intelligence technology, is increasingly utilized in medicine. To facilitate and expedite patients’ navigation to the appropriate department, we conducted an outpatient triage evaluation of ChatGPT. For this evaluation, we posed 30 highly representative and common outpatient questions to ChatGPT and scored its responses using a panel of five experienced doctors. The consistency of manual triage and ChatGPT triage was assessed by five experienced doctors, and statistical analysis was performed using the Chi-square test. The expert ratings of ChatGPT’s answers to these 30 frequently asked questions revealed 17 responses earning very high scores (10 and 9.5 points), 7 earning high scores (9 points), and 6 receiving low scores (8 and 7 points). Additionally, we conducted a prospective cohort study in which 45 patients completed forms detailing gender, age, and symptoms. Triage was then performed by outpatient triage staff and ChatGPT. Among the 45 patients, we found a high level of agreement between manual triage and ChatGPT triage (consistency: 93.3–100%, *p*<0.0001). We were pleasantly surprised to observe that ChatGPT’s responses were highly professional, comprehensive, and humanized. This innovation can help patients win more treatment time, improve patient diagnosis and cure rates, and alleviate the pressure of medical staff shortage.

## Introduction

Recently, the National Bureau of Statistics of China reported that there were over 8.42 billion outpatient visits in the country in 2022 (1). With such a large volume of patients seeking medical attention, effective triage becomes paramount for efficient and accurate diagnosis and treatment. Correct triage is crucial for the effective management of patients’ health conditions (2, 3). Traditional manual triage methods are often influenced by the experience and seniority of medical staff (4). However, intelligent triage systems, such as those based on AI, eliminate these potential biases (5). Studies have shown that smart phone triage applications can reduce the error rate in triage decisions, shorten consultation times, and help relieve the pressure on medical staff (5). Despite these advancements, the interaction mode of mobile App triage is still relatively fixed and may not provide personalized feedback to patients. In recent years, AI systems based on Chat Generation Pre-Training (ChatGPT) have gained significant attention and are increasingly being applied in healthcare settings (6). However, the application of ChatGPT in outpatient triage has not been fully explored. Therefore, this study aims to evaluate the utility of ChatGPT in outpatient triage. We hope to demonstrate the potential of ChatGPT to enhance triage accuracy, speed, and patient satisfaction, while also reducing the workload on medical staff.

## Methods

This study employed a retrospective cohort study and a prospective cohort study.

Retrospective Cohort Study: In March 2023, we conducted a random sampling of 30 outpatient medical records out of the vast pool of 100,000, spanning across the departments of Internal Medicine, Surgery, Gynecology, Pediatrics, and the Emergency Department at the First Affiliated Hospital of Gannan Medical University. The symptoms (Supplementary Figure S1) of these 30 cases were representative of common clinical symptoms encountered in clinical practice (7, 8). ChatGPT was used to answer 30 corresponding questions, and the responses were then scored by 5 experts. All 30 responses were independently assessed by 5 experts and given a score, which was ultimately averaged to ensure accuracy and consistency.

Prospective Cohort Study: We provided a form with age, gender, and symptoms, and randomly assigned 45 outpatients to fill out. Based on the tabular information, triage was performed both manually and using ChatGPT. The consistency of manual and ChatGPT triage was evaluated by 5 experts, and statistical analysis was performed using the Chi-square test. The manual triage personnel included professionally trained nurses and healthcare-related personnel. The assessments of the 5 experts are independent.

The 5 experts were all doctors who had worked in tertiary general hospitals for more than 5 years and held the qualification of attending physicians. They worked in departments such as respiratory medicine, hematology, oncology, pediatrics, and general surgery. They are assessed independently, first answering questions based on their own expertise and then evaluating ChatGPT’s responses. The independent evaluation by experts was based on the following principles: 1. Accuracy of ChatGPT triage; 2. Clarity of language expression; 3. Degree of first aid awareness; 4. Service attitude.

This study was conducted anonymously and without compensation, and was approved by the Ethics Committee of Ruijin People’s Hospital (approval No. 2023002). ChatGPT-3.5 was used in the study.

## Results

**Figure 1:**
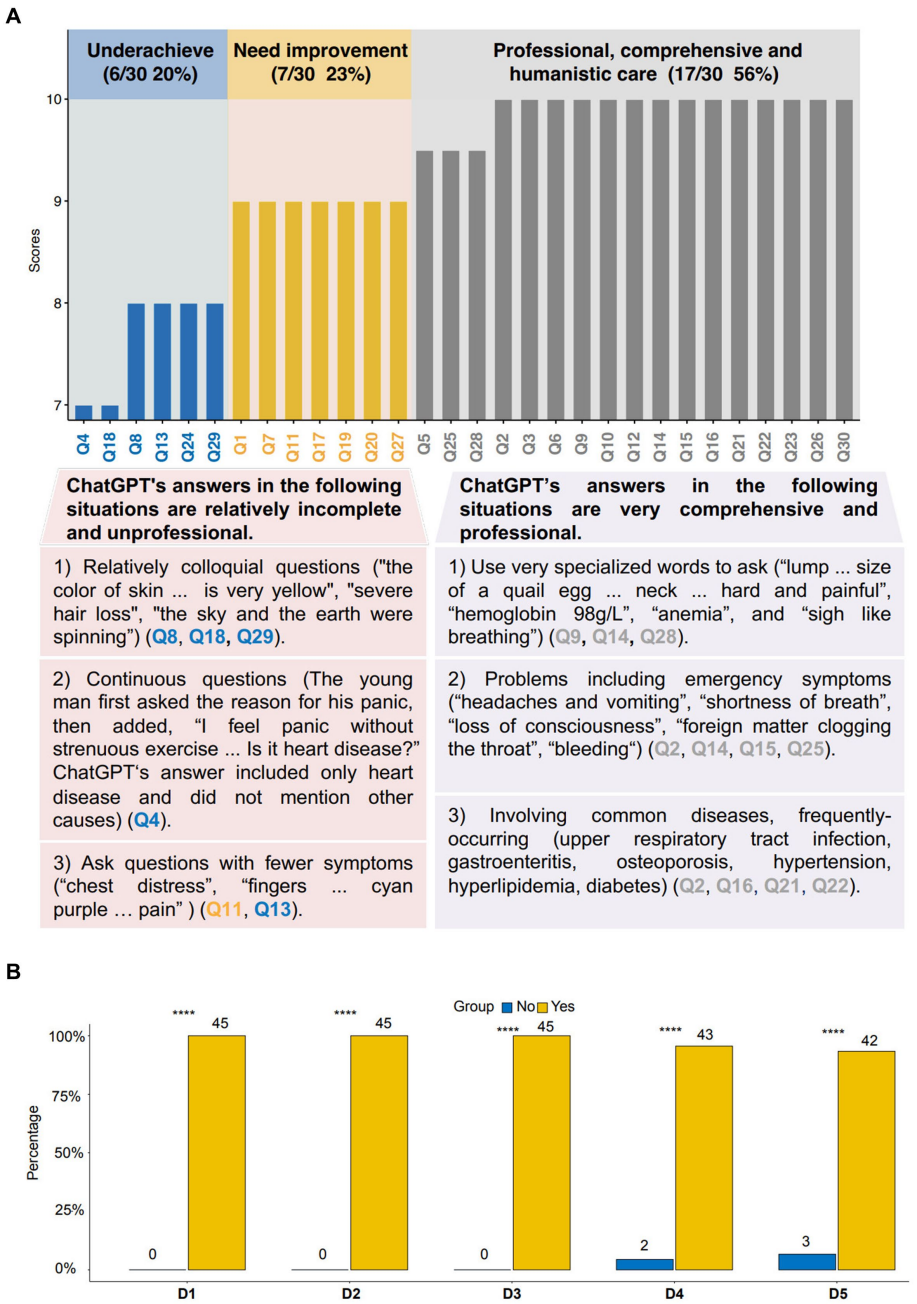
The reviewer’s evaluation of ChatGPT outpatient triage. **(A)** The reviewer’s rating of ChatGPT’s answers. **(B)** Reviewers assessed the consistency of manual and ChatGPT triage. Q, Questions. D, Doctor. Yes: ChatGPT triage in the same number as manual triage. No: Number of ChatGPT triage was inconsistent with manual triage. ****p<0.0001. The chi-square test was used in this study.

The retrospective cohort study revealed that among the 30 answers reviewed by 5 experts, 17 received high scores (10 and 9.5 points), 7 received relatively high scores (9 points), and 6 received relatively low scores (8 and 7 points; Figure 1A). The 17 high-scored answers reflect comprehensive and professional analysis, hierarchical diagnosis and treatment systems, first aid concepts, and humanization. The 7 high-scored answers are generally professional and comprehensive but have room for improvement. The 6 relatively low-scored answers are relatively incomplete and unprofessional. These are shown in Figure 1A, Table 1, and Supplementary Table 1.

**Table 1:** ChatGPT’s evaluation of outpatient symptoms and the doctor’s comments on ChatGPT’s answer.

n	Questions	ChatGPT answer	Score	Evaluation
1	I’m a woman, 26 years old. I’ve been coughing since I caught a cold. Which department should I go to?	It is recommended that you go to the respiratory doctor. Cough is a common symptom of a cold, but if it lasts for too long, it may mean that infection has caused respiratory infections or other related issues. A respiratory doctor can determine the cause by checking your symptoms, physical examinations and necessary laboratory examinations, and formulate the corresponding treatment plan for you.	9	The answer is very professional. The terminology it uses is almost the same as that of the respiratory doctor.
2	The patient is my daughter, 10 years old. She started fever, headache, and vomiting at night. What diseases do we need to consider and which department should we go to see?	If your daughter starts fever, headache, and vomiting at night, it may indicate that she is experiencing some health problems. These symptoms may be related to many diseases, including colds, influenza, viral gastroenteritis or other diseases. It is recommended that you take her to see a doctor as soon as possible to determine the severity of the problem and the best treatment. You can take her to see a pediatrician or a family doctor. Doctors may conduct some examinations to determine the cause and give corresponding treatment suggestions. In the process of waiting for the clinic, you can let her rest, keep moisture intake, control body temperature, and pay attention to any new symptoms. If your daughter’s symptoms become more serious, such as symptoms such as shortness of breathing, loss of consciousness, and neck stiffness, please immediately send it to the emergency department of the hospital nearby.	10	It can not only analyze the symptoms in a comprehensive manner, but also analyze the possible serious situation and provide accurate suggestions.

n, problem number; Score: 0 to 10, 0 is the lowest, 10 is the highest; Evaluation: Summary of the comments of multiple doctors on ChatGPT’s answers. Table 1 is just two of the questions answered by ChatGPT; the remaining 28 questions are shown in Supplementary Table 1.

The prospective cohort study revealed that among these 45 outpatients, five specialists considered manual triage to be particularly consistent with ChatGPT triage. We found that 3 reviewers thought that the consistency of manual and ChatGPT triage was 100% (*p*<0.0001; Figure 1B), 1 reviewer thought that the consistency was 95.6% (*p*<0.0001; Figure 1B), and 1 reviewer thought that the consistency was 93.3% (*p*<0.0001; Figure 1B).

Overall, the results indicated that ChatGPT’s answers provided accurate and professional triage information to patients without providing misinformation or harmful information to patients.

## Discussion

Outpatient triage is a necessary service in many parts of the world, especially where primary care systems are weak and primary care physicians work short weeks (9). Outpatient triage can improve treatment efficiency, reduce hospital queuing time, and improve medical efficiency, better meeting patients’ medical needs (3). With a shortage of medical staff, short consultation times for primary care physicians and non-24-h outpatient triage staff (9), we needed a tool that could help patients triage in real time. Traditional websites and apps can help triage patients, but the disadvantage is that the operation is complex, the information is broad and confusing, and cannot provide instant personalized feedback (10). However, this study shows that manual triage is highly consistent with ChatGPT triage and can provide professional, comprehensive, and humanized triage. ChatGPT can provide an interactive experience closer to human conversation, providing instant personalized feedback. Furthermore, ChatGPT possesses certain constraints, encompassing potential biases, reliability issues, privacy apprehensions, and ethical considerations surrounding its utilization (11, 12). Consequently, it is imperative to consistently update, train, and enhance ChatGPT to guarantee the security and credibility of the information it provides. Additionally, ethical frameworks ought to be formulated to tackle ethical quandaries stemming from its application in healthcare. This study lacks a large multicenter study. For future inquiries, it is envisaged that we shall amass specimens from numerous hospitals, regions, and centers, thereby augmenting the sample size and executing a multicenter study. Additionally, the triage of outpatient patients utilizing other AI models will be evaluated and contrasted with the triage by ChatGPT, providing a comprehensive comparison of their respective efficiencies. In the future, we hope that ChatGPT triage can be operated in healthcare facilities, so that patients can have a more convenient and faster medical experience. It is anticipated that ChatGPT will attain broader adoption in the medical sphere in the foreseeable future.

## Data availability statement

The original contributions presented in the study are included in the article/Supplementary material, further inquiries can be directed to the corresponding authors.

## Author contributions

XL: Conceptualization, Data curation, Formal analysis, Investigation, Methodology, Supervision, Validation, Writing – original draft, Writing – review & editing. RL: Conceptualization, Data curation, Formal analysis, Investigation, Methodology, Supervision, Validation, Writing – original draft, Writing – review & editing. CY: Data curation, Formal analysis, Methodology, Writing – original draft. ZG: Data curation, Methodology, Writing – original draft. YY: Data curation, Formal analysis, Supervision, Writing – original draft. XZ: Data curation, Formal analysis, Writing – original draft. JL: Data curation, Formal analysis, Writing – original draft. LL: Data curation, Formal analysis, Writing – original draft. HJ: Conceptualization, Data curation, Formal analysis, Funding acquisition, Investigation, Methodology, Project administration, Resources, Software, Supervision, Validation, Visualization, Writing – original draft, Writing – review & editing. WZ: Conceptualization, Data curation, Formal Analysis, Funding acquisition, Investigation, Methodology, Project administration, Resources, Software, Supervision, Validation, Visualization, Writing – original draft, Writing – review & editing. CW: Conceptualization, Data curation, Formal analysis, Investigation, Methodology, Supervision, Validation, Writing – original draft, Writing – review & editing. YL: Conceptualization, Data curation, Formal Analysis, Funding acquisition, Investigation, Methodology, Project administration, Resources, Software, Supervision, Validation, Visualization, Writing – original draft, Writing – review & editing.
