# Supplementary figures and images for: Assessing the utility of artificial intelligence throughout the triage outpatients: a prospective randomized controlled clinical study
Source: Front Public Health. 2024 May 30;12:1391906. doi: 10.3389/fpubh.2024.1391906 (PMC11171710; doi:10.3389/fpubh.2024.1391906)

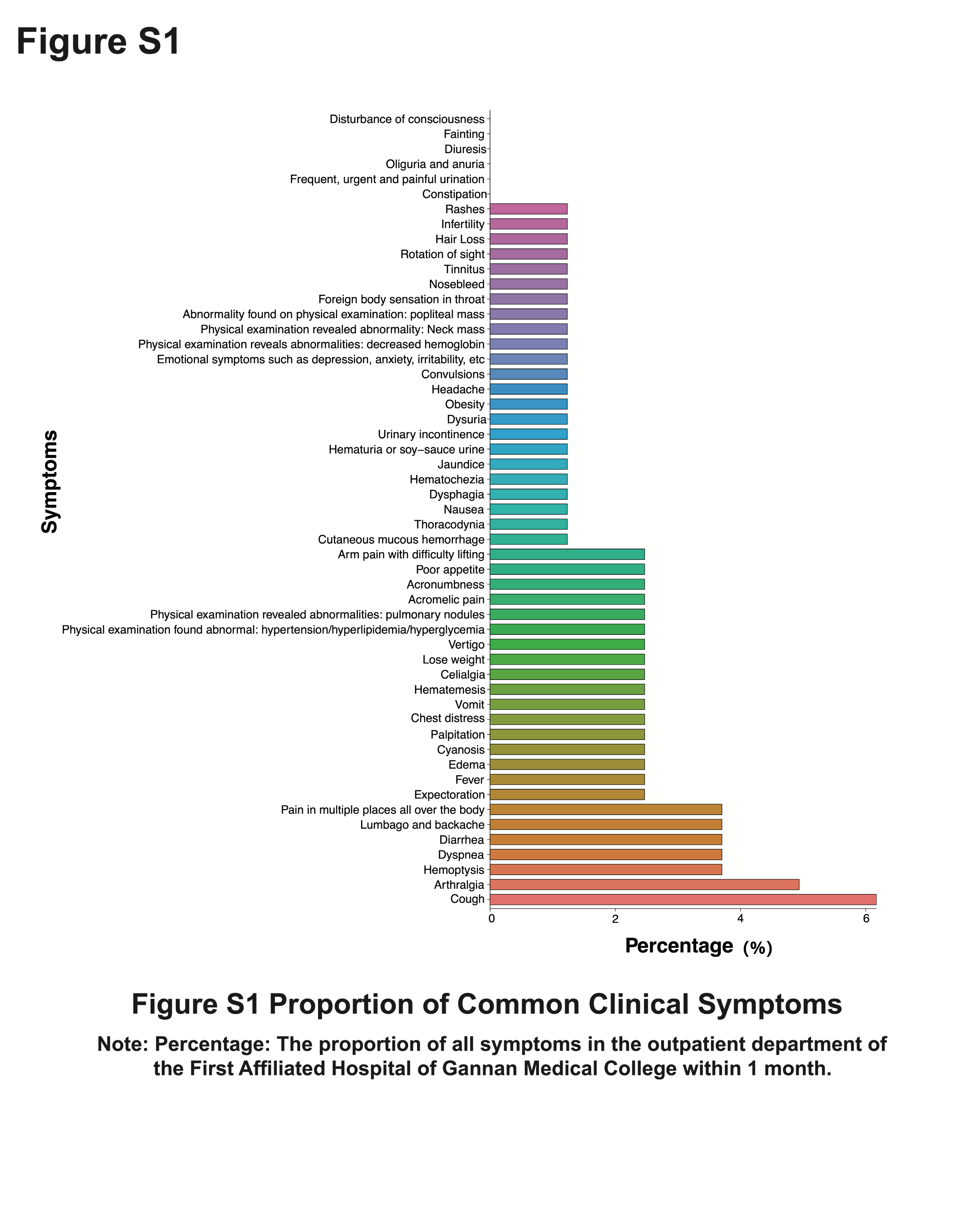

Supplement: Supplementary file 2 [file Image_1.PNG]
